# Multiparametric MRI radiomics for predicting disease-free survival and high-risk histopathological features for tumor recurrence in endometrial cancer

**DOI:** 10.3389/fonc.2024.1406858

**Published:** 2024-08-02

**Authors:** Mary Renton, Mina Fakhriyehasl, Jessica Weiss, Michael Milosevic, Stephane Laframboise, Marjan Rouzbahman, Kathy Han, Kartik Jhaveri

**Affiliations:** ^1^ The Joint Department of Medical Imaging, University Hospital Network, University of Toronto, Toronto, ON, Canada; ^2^ Department of Biostatistics, University Hospital Network, Toronto, ON, Canada; ^3^ Department of Radiation Oncology, University Hospital Network, Toronto, ON, Canada; ^4^ Department of Gynecologic Oncology, University Hospital Network, Toronto, ON, Canada; ^5^ Department of Laboratory Medicine and Pathobiology, University Hospital Network, Toronto, ON, Canada

**Keywords:** endometrial cancer, high-risk endometrial cancer, radiomics, MRI radiomics, disease-free survival

## Abstract

**Background:**

Current preoperative imaging is insufficient to predict survival and tumor recurrence in endometrial cancer (EC), necessitating invasive procedures for risk stratification.

**Purpose:**

To establish a multiparametric MRI radiomics model for predicting disease-free survival (DFS) and high-risk histopathologic features in EC.

**Methods:**

This retrospective study included 71 patients with histopathology-proven EC and preoperative MRI over a 10-year period. Clinicopathology data were extracted from health records. Manual MRI segmentation was performed on T2-weighted (T2W), apparent diffusion coefficient (ADC) maps and dynamic contrast-enhanced T1-weighted images (DCE T1WI). Radiomic feature (RF) extraction was performed with PyRadiomics. Associations between RF and histopathologic features were assessed using logistic regression. Associations between DFS and RF or clinicopathologic features were assessed using the Cox proportional hazards model. All RF with univariate analysis p-value < 0.2 were included in elastic net analysis to build radiomic signatures.

**Results:**

The 3-year DFS rate was 68% (95% CI = 57%-80%). There were no significant clinicopathologic predictors for DFS, whilst the radiomics signature was a strong predictor of DFS (p<0.001, HR 3.62, 95% CI 1.94, 6.75). From 107 RF extracted, significant predictive elastic net radiomic signatures were established for deep myometrial invasion (p=0.0097, OR 4.81, 95% CI 1.46, 15.79), hysterectomy grade (p=0.002, OR 5.12, 95% CI 1.82, 14.45), hysterectomy histology (p=0.0061, OR 18.25, 95% CI 2.29,145.24) and lymphovascular space invasion (p<0.001, OR 5.45, 95% CI 2.07, 14.36).

**Conclusion:**

Multiparametric MRI radiomics has the potential to create a non-invasive *a priori* approach to predicting DFS and high-risk histopathologic features in EC.

## Introduction

Endometrial cancer (EC) is the sixth most common cancer in women worldwide, with 417,000 new cases and 97,000 deaths in 2020 (1). Endometrial cancer is classified into two histopathological subtypes type I- endometrioid carcinoma accounting for 80% and type II-non-endometrioid subtypes including serous, clear cell and carcinosarcoma, which confers a worse prognosis (2). Whilst traditionally, further risk stratification is achieved through histologic grade and staging, the advances in genetic profiling in EC have resulted in a shift towards a molecular classification of EC based on four distinct genomic subgroups (2, 3).

Initial disease characterisation determines appropriate therapeutic strategies such as surgery, radiation, chemotherapy, hormonal and immunotherapy. Early-stage disease is primarily treated with hysterectomy and bilateral salpingo-oophrectomy, with adjuvant therapies traditionally reserved for intermediate-high risk or advanced disease (2).

MRI and endometrial biopsy have an important role in the diagnosis and evaluation of EC.

However, there are limitations to these assessments, including the inability to radiologically assess important histopathologic features like lymphovascular space invasion (LVSI) and sampling error or bias inherent to biopsy. There remain challenges to predicting long-term outcomes and disease-free survival (DFS). Currently, the critical prognostic factors for EC include tumour grade, histological subtype, genomic subtype, deep myometrial invasion (DMI), LVSI and lymph node metastases (LNM) (2, 3). Many of the prognostic features in the International Federation of Gynecology and Obstetrics (FIGO) classification for staging endometrial cancer rely on histopathology assessment of post-hysterectomy surgical specimens (2). Optimising preoperative radiological assessment could decrease reliance on and potentially circumvent surgical staging and therapeutic lymphadenectomy.

Radiomics is the process of computational extraction of vast amounts of quantitative voxel metrics from a segmented region of interest (ROI) on diagnostic images, which undergo statistical techniques to extract spatiotemporal features, thought to reflect the underlying pathophysiology and tumour phenotype (4). Radiomics has the potential to create a non-invasive *a priori* approach to personalised patient risk-stratification and refine the clinical decision-making process. Despite radiomic advances in other oncological areas, there remains a relative dearth of literature with regards to the prediction of high-risk histopathologic features for tumour recurrence in EC (5). In particular, there are a limited number of publications establishing the role of MRI radiomics in endometrial cancer DFS (6–9).

Thus, we aimed to explore a multiparametric MRI radiomics model predictive for DFS and high-risk histopathologic features associated with tumour recurrence in endometrial cancer.

## Methods

**Figure 1:**
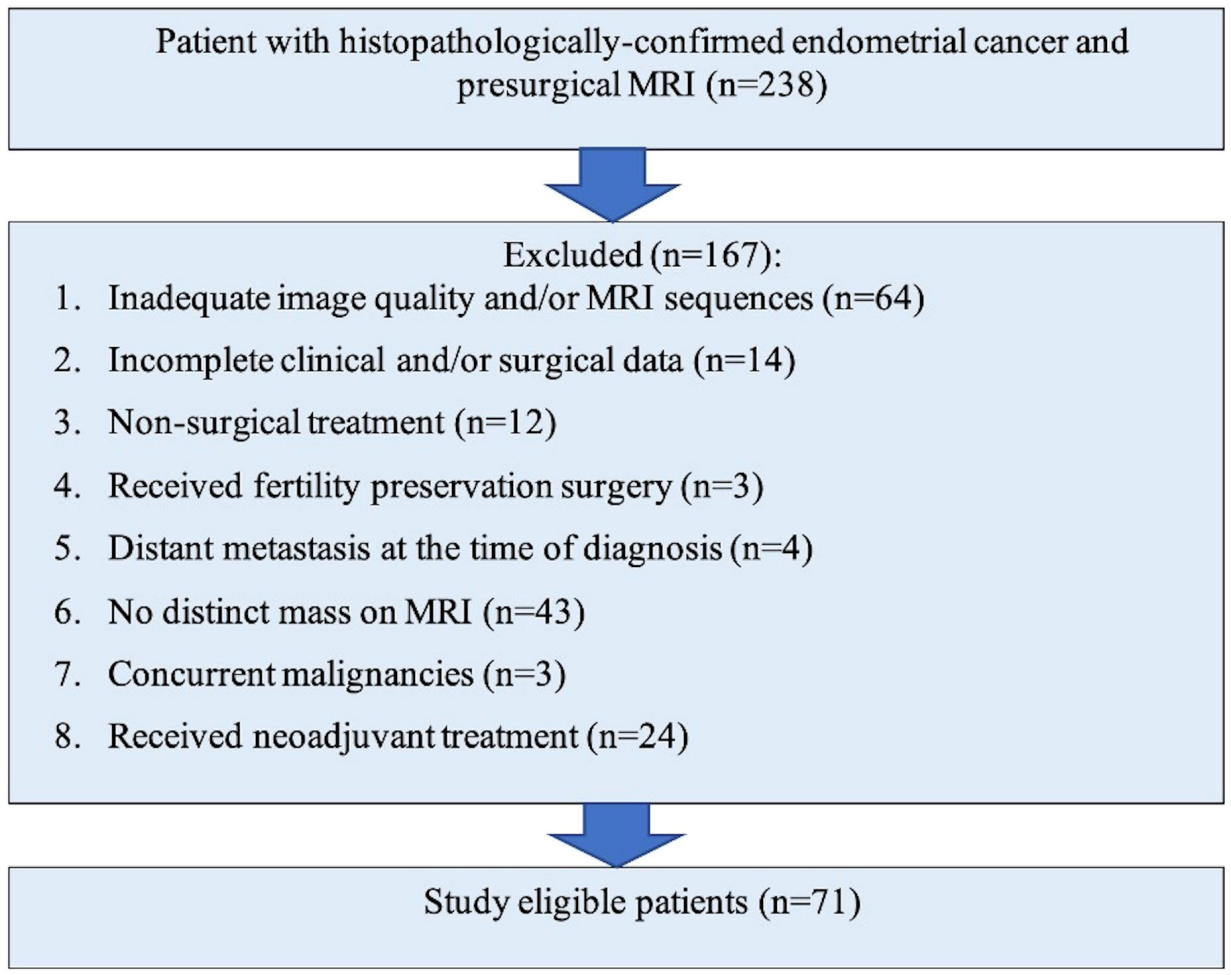
Flowchart of study population selection and exclusion criteria.

This was a retrospective study in a large Canadian tertiary referral institute with approval from our HIPPA-compliant institutional research and ethics board with waiver of informed consent. The initial surgical database search over a ten-year period (2010 – 2020) identified 238 consecutive patients with endometrial cancer who underwent MRI of which 167 were excluded due to occult disease on MRI, inadequate imaging, incomplete surgical data, distant metastases, concurrent neoplasm at diagnosis, fertility sparing surgery and preoperative neoadjuvant treatment (**Figure 1**). The final study cohort comprised of 71 patients who had a preoperative MRI and underwent operative management for histopathology-proven endometrial cancer at our institution. Patients with all histological subtypes and grade of endometrial cancer were included.

Patient characteristics and data were collected from the institutional electronic patient record (EPR) system.

### Baseline clinical characteristics

Patient age, date of diagnosis (defined as the biopsy date) and the date and type of surgery.

### Histopathology data

All histopathology specimens were processed and evaluated in accordance with a standard institutional protocol by an experienced gynaecologic pathologist. With grossly identifiable endometrial mass, one section per cm of the tumour is taken, including at least one full thickness section at the deepest point of myometrial invasion. If there is no gross mass, two full thickness sections, one anterior and one posterior endometrium and then the entire endometrium with djacent 2-3 mm of myometrium is submitted for microscopic examination. The pathology reports from the diagnostic endometrial biopsies and surgical resections were reviewed and evaluated according to the revised 2009 FIGO criteria for EC (10). The following data was recorded: tumour size, histopathology subtype (endometrioid or non-endometrioid), low/high histologic tumour grade (1/2 + 3, respectively), DMI, surgical resection margin, LVSI (positive, negative or indeterminate), lymph node involvement (number and location), tumour nodes and metastases (TNM) staging, extrauterine disease and immunohistochemistry mismatch repair protein (IHC MMR, intact or abnormal). DMI was classified as invasion 50% of the depth of myometrium. LVSI was defined as tumour cells in an endothelial-lined space distinct from the invasive border of the tumour mass.

### Clinical outcomes

The date of the last clinical follow-up and the disease status at final follow-up was recorded. DFS was defined as the time from surgery until disease recurrence was detected (radiologically, clinically or biochemically) or until the last follow-up, if the patient remained disease-free. Disease recurrence included local and/or metastatic disease. Where applicable, the date of disease recurrence, type of disease recurrence (local, pelvic/para-aortic nodes, distant metastasis) and date of death was recorded. Patient follow-up was as per institutional standard of care surveillance guidelines, including clinical examination, and where applicable cancer antigen-125 (CA-125), MRI and/or computed tomography (CT).

### Image acquisition, segmentation and feature extraction

The preoperative MRI examinations were performed on 1.5T or 3T systems (Magnetom Avanto or Verio, Siemens Healthineers, Erlangen, Germany) with a standardised institutional protocol (**Table 1**), including supine patient positioning, the intravenous administration of 20mg Hyoscine-butylbromide (Buscopan, Boehringer Ingelheim, Canada) and intravenous gadolinium contrast media. The following three sequences were selected for segmentation based on their robust signal to noise ratios: T2 weighted, apparent diffusion coefficient (ADC), and T1 weighted-dynamic contrast enhanced series (arterial, venous and delayed venous phases). MRI images were retrieved from our institutional Picture Archive and Communication System (PACS) and exported as anonymised DICOM images to an open-source DICOM viewer (3D slicer, version 4.11) (11).

**Table 1:** Summary of sample technical parameters for our institutional routine endometrial cancer MRI protocol, limited to the sequences used for segmentation and analysis.

	T2w (axial)	DWI/ADC (axial)		DCE T1w (axial)
Field Strength (Telsa)	1.5/3.0	1.5	3.0	1.5/3.0
Sequence type	Turbo spin echo	Echo-planar imaging		3D GRE
TR	3990	4300	5300	3.51
TE	94	72	70	1.4
Matrix size	320 x 320	192 x 157	192 x 144	320 x 256
Field of view (mm)	200 x 200	300 x 300	340 x 340	240 x 240
Slice thickness (mm)	4	4	4	4
Flip angle (degrees)	140	9	–	9
Interslice gap	1	1	1	0
B values	–	100, 400, 800		–

ADC, apparent diffusion coefficient; DCE, dynamic contrast enhancement; DWI, diffusion weighted images; GRE, gradient recalled echo; TE, echo time; TR, repetition time.

A radiologist, with 5-years’ experience, performed manual whole-tumour segmentation on all axial images of the relevant sequences wherein the tumour was identified; in the event the axial images were not suitable the coronal or sagittal plane images were used for contouring. At the time of segmentation, the Radiologist was blinded to the patient details, clinicopathologic features and disease outcomes but was aware of the diagnosis of endometrial cancer. Radiomic features (RF) were extracted from the ROIs on the contoured images using open-source software (Pyradiomics plugin to 3D Slicer, version 1.30) (11).

**Figure 2:**
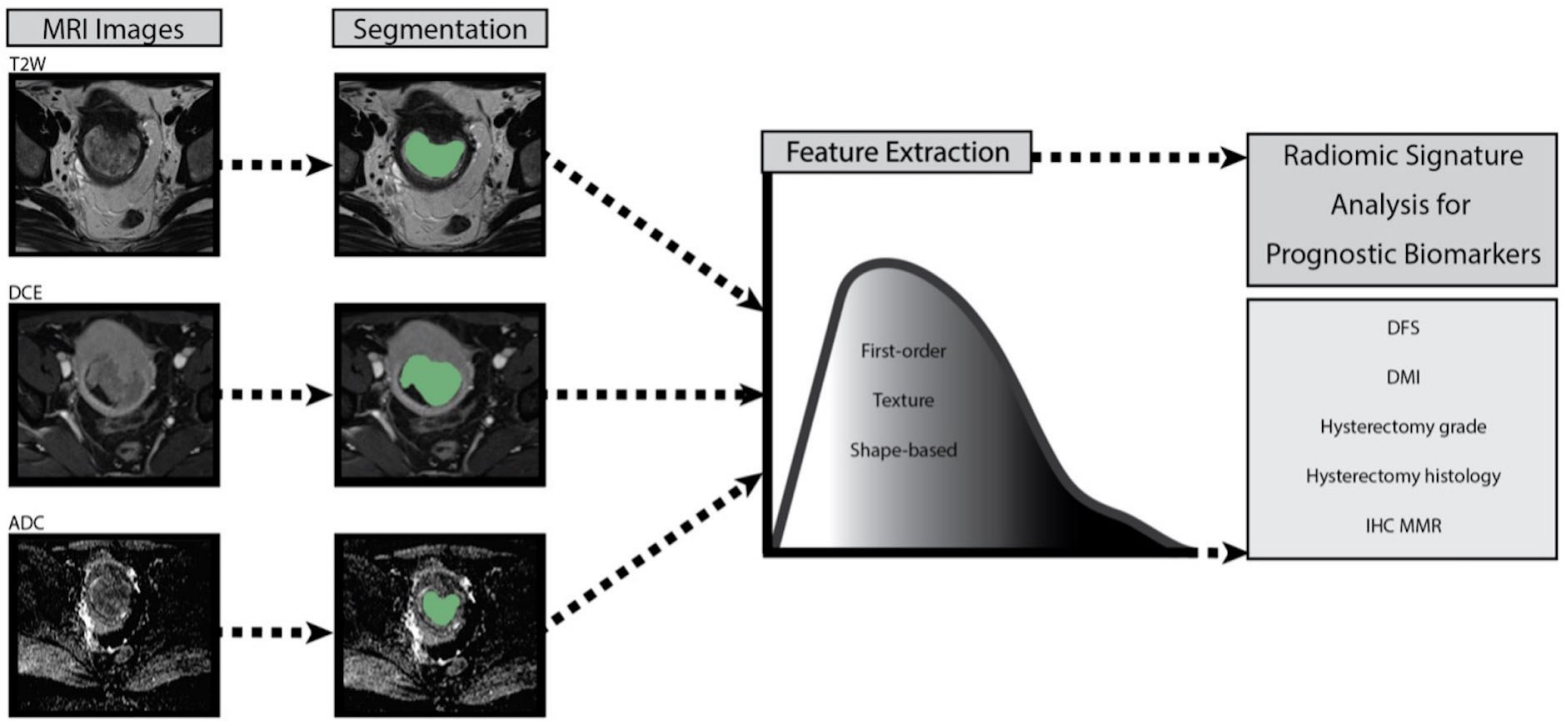
Process schematic of image processing and radiomic feature extraction for each patient with endometrial cancer. T2W T2 weighted, DCE dynamic contrast enhanced, ADC apparent diffusion coefficient, DFS disease-free survival, DMI deep myometrial, IHC MMR immunohistochemistry mismatch repair protein.

Source images were not resampled or filtered, and the bin-width was fixed at 25. Feature classes are listed in the software release notes, in accordance with the imaging biomarker standardisation initiative (IBSI) (12). The image processing and RF are summarised in the process schematic (**Figure 2**). The parameters were dichotomised for radiomics analysis as shown in **Table 2**.

**Table 2:** Summary of clinicopathological data.

Patient and Tumour Characteristics	Number (%)
Biopsy grade	n=54
grade 1	28 (52)
grade 2	11 (20)
grade 3	15 (28)
Biopsy histology	n=70
endometrioid adenocarcinoma	46 (66)
adenocarcinoma not otherwise specified	4 (6)
serous	4 (6)
carcinosarcoma	7 (10)
clear cell	1 (1)
mixed/other	8 (11)
IHC MMR	n=43
MMR intact	26 (60)
MMR abnormal	17 (40)
Hysterectomy grade	n=59
grade 1	21 (36)
grade 2	12 (20)
grade 3	26 (44)
Hysterectomy histology	n=70
endometrioid adenocarcinoma	49 (70)
serous	6 (9)
carcinosarcoma	9 (13)
clear cell	1 (1)
mixed/other	5 (7)
Tumour size (cm)	n=48
Mean (sd)	6.0 (2.9)
Range	0.1-14.5
Lymphovascular space invasion (LVSI)	n=71
no LVSI	32 (45)
LVSI	36 (51)
indeterminate	3 (4)
Deep myometrial invasion	n=71
≤50%	27 (38)
>50%	44 (62)
Type of surgery	n=70
hysterectomy bilateral salpingo-oophorectomy (hyst BSO)	13 (19)
hyst BSO + pelvic nodes	13 (19)
hyst BSO + pelvic nodes, omentectomy	24 (34)
hyst BSO, pelvic + paraaortic nodes	2 (3)
hyst BSO, pelvic + paraaortic nodes, omentectomy	12 (17)
other	6 (9)
Cervical involvement	n=70
yes	27 (39)
no	43 (61)
Adnexal involvement	n=69
yes	11 (16)
no	58 (84)
Surgical Margin	n=28
positive	7 (25)
negative	21 (75)
FIGO Stage	n=71
1a	9 (13)
1b	26 (37)
2	15 (21)
3a	6 (8)
3c	15 (21)
Positive pelvic nodes	n=54
0	40 (74)
1	5 (9)
2	6 (11)
3	1 (2)
5	1 (2)
8	1 (2)
Positive paraaortic nodes	n=23
0	20 (87)
2	1 (4)
4	1 (4)
5	1 (4)
Postoperative chemoradiotherapy	n=71
Radiation	69 (97)
Chemotherapy	34 (48)
Combined chemoradiotherapy	32 (45)

Hyst BSO, Hysterectomy + bilateral salpingoophorectomy; DMI, deep myometrial invasion; IHC MMR, immunohistochemistry mismatch repair protein; LVSI, lymphovascular space invasion.

### Statistical analysis

Patient characteristics were summarised using the mean (standard deviation, sd) for continuous variables and using counts and percentages for categorical variables. Associations between RF and histopathologic features were assessed using logistic regression.

Associations between DFS and RF or clinicopathologic features were assessed using univariate Cox proportional-hazards model. All RF with p-value < 0.2 on univariate analysis were included in elastic net analysis to build radiomic signatures. Elastic net regularisation was used as the method for feature selection for both Cox proportional-hazards models, and logistics regression models. Elastic net was selected as the number of predictors is higher than the number of samples used. For each model the lamba value was selected as the value that gives minimum mean cross-validated error, and an alpha value of 0.5 was used for all models as to balance both the LASSO and Ridge regression aspects. The fit of the Cox proportional-hazards model was evaluated using the concordance index, and for the logistics regression models model fit was assessed with the Area Under the Curve (AUC).

## Results

### Baseline clinicopathology data

Patients included in the final study cohort (n=71) had a mean age of 58.9 +/- 12.8 years old (range 35-84). Their clinicopathological data are summarised in **Table 2**. The mean time from date of biopsy to surgery was 12.4 weeks (± 12.3 weeks, range 0-75.6 weeks).

### High-risk histopathologic features for tumour recurrence and radiomic features

Overall, 107 radiomic features (RF) from seven feature classes were extracted from each segmentation image. These features include first-order histogram statistics (18 features), shape descriptors (14 features) and texture features calculated from the gray level co-occurrence matrix (glcm, 24 features), gray level dependency matrix (gldm, 14 features), gray level run length matrix (glrlm, 16 features), gray level size zone matrix (glszm, 16 features) and neighbouring gray - one difference matrix (ngtdm, 5 features). The univariate Cox proportional logistic regression models demonstrated multiple significant associations between individual RF and all high-risk histopathologic features (**Supplementary Table 1**).

Elastic net radiomic signatures were created for DFS, DMI, IHC MMR, hysterectomy grade, hysterectomy histology and LVSI, using a range of 2 to 9 RF. Significant correlations (p<0.05) were established between the elastic net signatures and all of the high-risk histopathologic features for tumour recurrence, except IHC MMR (p=0.074). The individual RF in each elastic net signature and their corresponding coefficients and significance are collated in **Table 3**.

**Table 3:** Elastic net signatures for high-risk histopathologic features for tumour recurrence and disease free survival, with the comprised individual radiomics features and their coefficients, and the elastic net signature associations.

Elastic net signature features	Coefficient	HR (95% CI)	p-value	Concordance index
DFS				
firstorder_Kurtosis	0.16	3.62 (1.94,6.75)	<0.001	0.696
shape_Flatness	1.15			
glrlm_LongRunHighGrayLevelEmphasis	0.001			
firstorder_Skewness	0.36			
firstorder_Range	2.00e-04			
ngtdm_Contrast	-8.94			
DMI (≤ 50% vs >50%)		OR (95% CI)	p-value	AUC
(Intercept)	1.01	4.81 (1.46,15.79)	0.010	0.679
gldm_DependenceNonUniformityNormalized	-8.21			
firstorder_InterquartileRange	-0.002			
glcm_Contrast	-0.025			
firstorder_RobustMeanAbsoluteDeviation	-6.38e-05			
glcm_SumEntropy	-0.09			
glcm_DifferenceVariance	-0.004			
ngtdm_Contrast	-0.01			
glcm_MaximumProbability	0.5			
ngtdm_Complexity	-8.656e-05			
Hysterectomy grade (1 vs 2/3)		OR (95% CI)	p-value	AUC
(Intercept)	-1.78	5.12 (1.82,14.45)	0.002	0.733
glszm_ZonePercentage	4.12			
gldm_SmallDependenceEmphasis	1.68			
gldm_DependenceNonUniformityNormalized	1.04			
ngtdm_Contrast	3.34			
firstorder_Minimum	0.002			
gldm_LargeDependenceHighGrayLevelEmphasis	-4.53e-06			
glszm_SizeZoneNonUniformity	-1.64e-04			
Hysterectomy histology (Endometrioid adenocarcinoma vs other)		OR (95% CI)	p-value	AUC
(Intercept)	2.98	6.25 (2.05,19.08)	0.001	0.700
glcm_Imc2	-0.69			
glcm_MCC	-0.34			
glcm_Imc1	6.25			
gldm_DependenceEntropy	-0.022			
glcm_SumAverage	-6.83e-04			
glcm_JointAverage	-0.001			
firstorder_InterquartileRange	-0.001			
firstorder_RobustMeanAbsoluteDeviation	-0.002			
shape_Flatness	-1.63			
glrlm_RunLengthNonUniformity	-7.205e-07			
firstorder_Variance	-2.063e-07			
gldm_LargeDependenceLowGrayLevelEmphasis	0.068			
IHC MMR (vs MMR abn)		OR (95% CI)	p-value	AUC
(Intercept)	0.08	3.89 (0.88,17.31)	0.074	0.696
glcm_Imc1	-7.71			
gldm_SmallDependenceHighGrayLevelEmphasis	-0.02			
LVSI (+indeterminate vs no LVSI)		OR (95% CI)	p-value	AUC
(Intercept)	-0.077	5.45 (2.07,14.36)	<0.001	0.775
shape_Flatness	-1.98			
glcm_InverseVariance	-3.98			
firstorder_Kurtosis	0.34			
glcm_Imc2	2.48			
glszm_ZonePercentage	0.38			
gldm_DependenceNonUniformityNormalized	2.04			
firstorder_Skewness	0.14			
shape_Maximum2DDiameterRow	-0.01			
glrlm_RunVariance	0.37			

AUC, area under the curve; DFS, disease-free survival; DMI, deep myometrial invasion; IHC MMR, immunohistochemistry mismatch repair protein; LVSI, lymphovascular space invasion.

### Clinical outcomes: DFS

The median follow up from the date of surgery was 2.8 years (range=0.2-9.4). The mean follow up period for patients without recurrence was 4.1 years (range: 0.6-9.4 years). The 3-year DFS rate was 68% (95% CI = 57%-80%). The 3 year local, regional, and distant recurrence rates were 18.9% (95%CI = 9.9%-30.2%), 13.2% (95%CI = 6.0%-23.3%) and 23.7% (13.6%- 35.4%) respectively. Almost all of the patients received postoperative pelvic radiotherapy (69, 97.2%) and almost half of the patients received chemotherapy (34, 47.9%). Of the patients receiving chemotherapy, 9 (26.5%) were palliative therapies.

There were no significant clinicopathological predictors of DFS: hysterectomy grade (p=0.14, HR 2.13 95% CI 0.77, 5.87), hysterectomy histology (p=0.42, HR 1.42, 95% CI 0.61, 3.28) and disease stage (p=0.66, HR 1.21 95% CI 0.51, 2.85). As further outlined in **Table 3**, a DFS elastic net signature was created based on 6 RF and was a strong predictor of DFS (p<0.001, HR 3.62, 95% CI 1.94, 6.75).

## Discussion

Our results show that multiparametric MRI radiomics could potentially be used preoperatively in patients with EC to predict DFS and important high-risk histopathologic features associated with tumour recurrence. We established a six-feature elastic net radiomic signature that was a strong predictor of DFS, while clinicopathologic features had no significant correlation with DFS. We established statistically significant individual RF for the high-risk histopathologic features and statistically significant elastic net MRI radiomic signatures specifically for DMI, hysterectomy grade, hysterectomy histology, and LVSI. This raises the possibility of inadequate clinicopathologic EC prognostication and the potential prognostic role for multiparametric MRI radiomics.

Our results demonstrating the role of MRI radiomics in predicting EC survival outcomes are in-keeping with a prospective study by Ytre-Hauge et al, in which high kurtosis in post-contrast T1WI was a good predictor of progression-free survival (HR 1.5, p<0.001) (6). In the same cohort, Jacob et al. reported an MRI radiomics model that predicted 5-year disease-specific survival (p < 0.001) (8). Whilst segmentation is a mainstay technique of radiomics literature, whole-tumor MRI RF have also been shown to determine significant predictors of progression-free survival (13). To the best of our knowledge, there is only one additional study to report MRI radiomics in predicting survival outcomes, which reports single sequence ADC metrics predicting disease recurrence and reduced survival (14). Our results contribute to the growing evidence in support of the use of MRI radiomics in the prognostication of EC survival outcomes.

In addition to DFS, we report the utility of MRI radiomics in predicting established high-risk histopathologic features for tumour recurrence in EC. LVSI represents microvessel tumour emboli and is one of the most important high-risk histopathologic features in EC, as it is considered a precursor to metastatic disease (3). In combination with similar contemporary studies, our demonstration of an MRI radiomics signature predictive for LVSI helps to establish the diagnostic predictive value of MRI radiomics for LVSI and its potential practical application in clinical decision-making (7, 15, 16). Ueno et al. and Zhang et al. also reported the predictive value of MRI radiomics in LVSI, DMI and high-grade tumours (15, 16). Long et al. in a cohort of n=184, showed T2WI and DCE T1WI-based radiomics had a strong predictive role for LVSI (AUC 0.93 95% CI: 0.875–0.991; sensitivity: 91.6%; specificity: 96.0%) (17).In contrast to these results, Bereby-Kahane et al, found a limited role for MRI radiomics in LVSI and high EC grade prediction (AUC of 0.59, sensitivity = 71%; specificity = 59%) and reported that tumour size (short axis 20mm) outperformed radiomics as their strongest predictor (18). Similar results were shown by Fasmer et al, as tumour size outperformed their MRI radiomic signatures for DMI and LMN (13). These discrepant findings may be due to their small sample sizes and, in the case of Fasmer et al, low numbers of high-risk surgicopathological features and a lack of multiparametric radiomic analysis.

Multiple studies have established robust MRI radiomic imaging biomarkers for LNM in EC (6, 9, 13, 15, 19, 20). Whilst we did not find a significant MRI radiomic signature for LNM, this is likely attributed to the low incidence of nodal positive disease in our cohort (n=14 positive pelvic nodes). The integration of clinical variables with radiomics modelling has shown MRI clinicoradiomics fusion models can also predict LNM in EC (19, 21). Xu et al. showed a combination of DCE T1WI RF, lymph node size and CA125, showed the best predictability for LNM and out-performed conventional radiologist MRI assessment (19). Yan et al. in a large (n=717) multicentre study with external validation, showed that clinicoradiomic fusion models have a strong diagnostic performance in predicting high-risk EC (AUCs of 0.75 and 0.85 in two validation groups) with applications in surgical decisions (22). Multiple additional studies, including our results, have also shown radiomics to have significant independent prediction in stratifying low or high-risk EC (6, 9, 13, 16, 18, 21–26). DMI is another important high-risk histopathologic feature for EC, with a developing role for MRI radiomics predictive models (6, 9, 13, 15, 16, 22, 25–29). Stanzione et al demonstrated radiomic-assisted MRI interpretation improved accuracy for DMI detection from 82% to 100% (p=0.48) (27). Less supportive results were reported by Otani et al, in a study of n=200 patients, their multiparametric MRI radiomics models predictive for DMI did not enhance the conventional radiologist MRI assessment (30). There are, however, multiple additional studies showing encouraging results for radiomic-assisted diagnostic performance compared with the conventional MRI evaluation in EC (19, 20, 24).

Over recent years, the advancement of molecular characterisation in EC, has resulted in a refinement of molecular prognostication based on the four distinct genomic subtypes proposed by The Cancer Genome Atlas (TCGA) (2, 3). In conjunction, radiomics has been shown to enhance EC genetic profiling in combined radiogenomic modelling that incorporates genomic tumour information (8, 9). We tested for one of the four TCGA subtypes and did not find significant individual RF or radiomic signature associations (p=0.074). Despite the increasing number of validated molecular biomarkers for EC, their clinical applications are still limited (3). This may be due in part to the limited availability for IHC MMR screening outside of tertiary centres. Similarly, there remains uncertainty regarding the transferability of MRI radiomics into clinical practice and the need for large scale validation studies is paramount. Integrating genomics, with clinicoradiomic fusion risk-stratification models could produce a more robust and clinically applicable approach to prognostication.

We must acknowledge several limitations of our study. Primarily, the retrospective single-institution study design resulting in a smaller patient cohort with the lack of validation may have also resulted in overfitting of the models. The limited sample number was too small to use model training and testing. The proportion of patients in our cohort with an IHC MMR status was low, limiting conclusions. While 3 years may be sufficient follow up time to predict an event in advanced stage (3-4) endometrial cancer, it is a relatively short time for follow up to predict DFS or survival in early-stage endometrial cancer. The analysis was initiated before the new FIGO 2023 staging system was published and therefore dichotomised into endometrioid vs non-endometrioid. Clinically, many hospitals have not yet adopted the new FIGO 2023 staging system and still categorizes endometrial cancer into endometrioid vs non-endometrioid. We utilised a tumour segmentation approach comprised of axial imaging of key pulse sequences only in majority of patients and even though all images wherein tumor was visible were contoured, theoretically in a heterogenous tumour this approach could possibly not be entirely representative. However, the literature comparing single slice and whole-tumour segmentation is currently inconclusive. Segmentations were performed by one radiologist, thus not allowing for assessment of reproducibility and stability of the radiomic features used for model development. Computational image preprocessing was not utilised, however image resampling remains a controversial process.

Additionally considering MRI data collection in this patient cohort was over a 10-year period, evolution in MRI technology could have also further contributed to data heterogeneity.

In conclusion, our results support the potential role for multiparametric MRI radiomics in EC to non-invasively predict DFS and critical high-risk histopathologic features for tumour recurrence, and thus optimise personalised patient care. However, despite the promising results, practical implementation in the clinical decision-making process has yet to come to fruition. To establish standardised non-invasive radiomics-aided preoperative prognostic modelling in EC, large-scale multi-institutional data sharing is likely necessary to facilitate comprehensive validation required for clinical translation and personalised patient management.

## Data availability statement

The raw data supporting the conclusions of this article will be made available by the authors, without undue reservation.

## Ethics statement

The studies involving humans were approved by University Health Network, Toronto, Canada. The studies were conducted in accordance with the local legislation and institutional requirements. The ethics committee/institutional review board waived the requirement of written informed consent for participation from the participants or the participants’ legal guardians/next of kin in accordance with institutional ethics review.

## Author contributions

MRe: Formal analysis, Methodology, Writing – original draft, Writing – review & editing. MF: Conceptualization, Data curation, Investigation, Software, Writing – original draft, Writing – review & editing. JW: Data curation, Formal analysis, Investigation, Validation, Writing – original draft, Writing – review & editing. MM: Methodology, Writing – original draft, Writing – review & editing. SL: Conceptualization, Methodology, Writing – original draft, Writing – review & editing. MRo: Data curation, Formal analysis, Writing – original draft, Writing – review & editing. KH: Conceptualization, Data curation, Formal analysis, Funding acquisition, Investigation, Methodology, Software, Supervision, Writing – original draft, Writing – review & editing. KJ: Conceptualization, Data curation, Formal analysis, Funding acquisition, Investigation, Methodology, Project administration, Resources, Software, Supervision, Validation, Visualization, Writing – original draft, Writing – review & editing.
