# Supplementary material for: Multiparametric MRI radiomics for predicting disease-free survival and high-risk histopathological features for tumor recurrence in endometrial cancer
Source: Front Oncol. 2024 Aug 2;14:1406858. doi: 10.3389/fonc.2024.1406858 (PMC11327158; doi:10.3389/fonc.2024.1406858)
Supplement: Supplementary file 1 [file Table_1.docx]

**Supplementary table 1.** Univariate analysis of high-risk histopathologic features for tumour recurrence, disease free survival and radiomic features. Only significant results with *p-value* <0.2 are shown. *DFS disease-free survival, DMI deep myometrial invasion, IHC MMR immunohistochemistry mismatch repair protein, LVSI lymphovascular space invasion.*

| **Features** |  |  |
| --- | --- | --- |
| **DFS** | **HR (95% CI)** | **p-value** |
| shape VoxelVolume | 1.00 (1.00,1.00) | 0.67 |
| **firstorder Kurtosis** | **1.65 (1.17,2.33)** | **0.0047** |
| **shape Flatness** | **23.41 (1.41,387.28)** | **0.028** |
| **glcm Idn** | **2e+13 (21.84,1.8e+25)** | **0.029** |
| **gldm LargeDependenceHighGrayLevelEmphasis** | **1.00 (1.00,1.00)** | **0.035** |
| **glrlm LongRunHighGrayLevelEmphasis** | **1.00 (1.00,1.00)** | **0.045** |
| firstorder Skewness | 2.82 (0.99,7.99) | 0.051 |
| firstorder Range | 1.00 (1.00,1.00) | 0.053 |
| ngtdm Contrast | 1.3e-11 (2.9e-24,57.60) | 0.092 |
| glszm HighGrayLevelZoneEmphasis | 1.00 (1.00,1.01) | 0.12 |
| ngtdm Complexity | 1.00 (1.00,1.00) | 0.12 |
| glszm SmallAreaHighGrayLevelEmphasis | 1.00 (1.00,1.01) | 0.15 |
| glrlm HighGrayLevelRunEmphasis | 1.00 (1.00,1.01) | 0.16 |
| glcm Autocorrelation | 1.00 (1.00,1.01) | 0.16 |
| gldm HighGrayLevelEmphasis | 1.00 (1.00,1.01) | 0.16 |
| glcm Idmn | 5e+21 (4.4e-10,5.6e+52) | 0.17 |
| firstorder Maximum | 1.00 (1.00,1.00) | 0.18 |
| glrlm ShortRunHighGrayLevelEmphasis | 1.00 (1.00,1.01) | 0.18 |
| **DMI (≤ 50% vs >50%)** | **OR (95% CI)** | **p-value** |
| **gldm DependenceNonUniformityNormalized** | **2.3e-10 (1.8e-18,0.03)** | **0.020** |
| **firstorder InterquartileRange** | **0.97 (0.95,1.00)** | **0.025** |
| **glcm DifferenceAverage** | **0.34 (0.13,0.88)** | **0.026** |
| **glcm Contrast** | **0.82 (0.69,0.98)** | **0.028** |
| **firstorder RobustMeanAbsoluteDeviation** | **0.94 (0.89,0.99)** | **0.029** |
| **glcm SumSquares** | **0.82 (0.68,0.98)** | **0.031** |
| **glcm SumEntropy** | **0.42 (0.19,0.93)** | **0.033** |
| **glcm JointEntropy** | **0.61 (0.38,0.96)** | **0.034** |
| **firstorder Entropy** | **0.39 (0.16,0.94)** | **0.036** |
| **glcm DifferenceVariance** | **0.62 (0.40,0.97)** | **0.037** |
| **firstorder MeanAbsoluteDeviation** | **0.96 (0.93,1.00)** | **0.037** |
| **glcm ClusterTendency** | **0.94 (0.89,1.00)** | **0.041** |
| **glcm Id** | **523.82 (1.25,2.2e+05)** | **0.042** |
| **gldm SmallDependenceHighGrayLevelEmphasis** | **0.96 (0.92,1.00)** | **0.042** |
| **glcm Idm** | **159.35 (1.17,2.2e+04)** | **0.043** |
| **firstorder Uniformity** | **1.5e+03 (1.18,1.9e+06)** | **0.045** |
| **gldm GrayLevelVariance** | **0.84 (0.71,1.00)** | **0.045** |
| **ngtdm Contrast** | **1.8e-15 (5.7e-30,0.58)** | **0.046** |
| **glrlm GrayLevelVariance** | **0.85 (0.72,1.00)** | **0.050** |
| **glcm DifferenceEntropy** | **0.30 (0.09,1.00)** | **0.050** |
| gldm SmallDependenceEmphasis | 2.7e-04 (6.8e-08,1.06) | 0.052 |
| glszm ZonePercentage | 9.2e-04 (7.9e-07,1.06) | 0.052 |
| firstorder Variance | 1.00 (1.00,1.00) | 0.053 |
| glcm MaximumProbability | 2.5e+03 (0.87,6.9e+06) | 0.054 |
| glrlm GrayLevelNonUniformityNormalized | 2.6e+03 (0.77,8.6e+06) | 0.058 |
| gldm DependenceVariance | 1.09 (1.00,1.20) | 0.060 |
| glcm InverseVariance | 9.2e+03 (0.65,1.3e+08) | 0.061 |
| ngtdm Complexity | 1.00 (1.00,1.00) | 0.068 |
| glcm JointEnergy | 2e+05 (0.26,1.5e+11) | 0.077 |
| glrlm RunLengthNonUniformityNormalized | 7.2e-03 (2.7e-05,1.88) | 0.082 |
| firstorder Range | 1.00 (1.00,1.00) | 0.092 |
| glrlm RunPercentage | 2.8e-03 (2.7e-06,2.79) | 0.095 |
| firstorder Maximum | 1.00 (1.00,1.00) | 0.10 |
| gldm LargeDependenceEmphasis | 1.01 (1.00,1.03) | 0.11 |
| **Hysterectomy Grade (1 vs 2/3)** | **OR (95% CI)** | **p-value** |
| **glszm ZonePercentage** | **1.1e+05 (34.94,3.5e+08)** | **0.0047** |
| **gldm SmallDependenceEmphasis** | **4.8e+05 (33.78,6.7e+09)** | **0.0073** |
| **gldm DependenceNonUniformityNormalized** | **1.3e+11 (693.56,2.5e+19)** | **0.0085** |
| **ngtdm Contrast** | **2.6e+20 (1.2e+05,5.4e+35)** | **0.009** |
| **firstorder Minimum** | **1.01 (1.00,1.01)** | **0.045** |
| glcm DifferenceAverage | 2.70 (0.97,7.51) | 0.056 |
| glcm Contrast | 1.18 (0.99,1.40) | 0.058 |
| ngtdm Strength | 13.05 (0.86,197.37) | 0.064 |
| glcm InverseVariance | 5.9e-05 (1.4e-09,2.37) | 0.072 |
| glcm DifferenceVariance | 1.47 (0.96,2.24) | 0.077 |
| glcm DifferenceEntropy | 3.40 (0.85,13.53) | 0.083 |
| glcm Idm | 6.4e-03 (2e-05,1.99) | 0.084 |
| glcm Id | 2e-03 (1.7e-06,2.36) | 0.085 |
| glrlm RunLengthNonUniformityNormalized | 258.12 (0.39,1.7e+05) | 0.094 |
| shape LeastAxisLength | 0.96 (0.91,1.01) | 0.13 |
| gldm LargeDependenceHighGrayLevelEmphasis | 1.00 (1.00,1.00) | 0.13 |
| glcm Idn | 3e-09 (1.7e-20,534.58) | 0.14 |
| shape Maximum2DDiameterRow | 0.98 (0.96,1.01) | 0.14 |
| glrlm RunPercentage | 489.60 (0.12,1.9e+06) | 0.14 |
| gldm SmallDependenceHighGrayLevelEmphasis | 1.03 (0.99,1.07) | 0.15 |
| gldm DependenceVariance | 0.93 (0.83,1.03) | 0.16 |
| glrlm ShortRunEmphasis | 1.5e+03 (0.06,3.8e+07) | 0.16 |
| shape SurfaceVolumeRatio | 8.76 (0.40,192.63) | 0.17 |
| firstorder 90Percentile | 1.00 (1.00,1.01) | 0.18 |
| firstorder RootMeanSquared | 1.00 (1.00,1.01) | 0.19 |
| firstorder Median | 1.00 (1.00,1.01) | 0.19 |
| firstorder Mean | 1.00 (1.00,1.01) | 0.19 |
| glszm SizeZoneNonUniformity | 1.00 (1.00,1.00) | 0.20 |
| **Hysterectomy histology**  (Endometrioid adenocarcinoma vs other) | **OR (95% CI)** | **p-value** |
| **gldm DependenceEntropy** | **0.20 (0.06,0.66)** | **0.0077** |
| **glszm ZoneEntropy** | **0.29 (0.12,0.74)** | **0.0097** |
| **glrlm RunEntropy** | **0.19 (0.05,0.72)** | **0.015** |
| **glcm Correlation** | **0.02 (7.6e-04,0.57)** | **0.022** |
| **glcm SumEntropy** | **0.36 (0.15,0.88)** | **0.025** |
| **glcm SumAverage** | **0.92 (0.86,0.99)** | **0.026** |
| **glcm JointAverage** | **0.85 (0.74,0.98)** | **0.026** |
| **firstorder Entropy** | **0.33 (0.12,0.88)** | **0.028** |
| **glszm GrayLevelNonUniformityNormalized** | **2.8e+07 (4.33,1.7e+14)** | **0.032** |
| **glrlm GrayLevelNonUniformityNormalized** | **1.1e+05 (2.60,4.7e+09)** | **0.033** |
| **glszm GrayLevelVariance** | **0.91 (0.84,0.99)** | **0.033** |
| **glcm MCC** | **0.01 (2.7e-04,0.74)** | **0.035** |
| **firstorder Uniformity** | **3.2e+04 (2.02,4.9e+08)** | **0.036** |
| **glcm Imc2** | **0.01 (2.7e-04,0.78)** | **0.038** |
| **glrlm LongRunHighGrayLevelEmphasis** | **1.00 (0.99,1.00)** | **0.039** |
| **firstorder MeanAbsoluteDeviation** | **0.96 (0.93,1.00)** | **0.042** |
| **firstorder Range** | **1.00 (0.99,1.00)** | **0.044** |
| **firstorder InterquartileRange** | **0.98 (0.96,1.00)** | **0.049** |
| **firstorder RobustMeanAbsoluteDeviation** | **0.95 (0.90,1.00)** | **0.049** |
| gldm LargeDependenceHighGrayLevelEmphasis | 1.00 (1.00,1.00) | 0.051 |
| glcm JointEntropy | 0.61 (0.36,1.01) | 0.053 |
| glszm HighGrayLevelZoneEmphasis | 0.99 (0.99,1.00) | 0.054 |
| glcm Autocorrelation | 0.99 (0.99,1.00) | 0.056 |
| gldm LowGrayLevelEmphasis | 4.6e+13 (0.20,1.1e+28) | 0.062 |
| glrlm HighGrayLevelRunEmphasis | 0.99 (0.99,1.00) | 0.063 |
| glszm SmallAreaLowGrayLevelEmphasis | 8.8e+15 (0.14,5.7e+32) | 0.063 |
| gldm HighGrayLevelEmphasis | 0.99 (0.99,1.00) | 0.063 |
| glrlm ShortRunHighGrayLevelEmphasis | 0.99 (0.99,1.00) | 0.067 |
| glrlm LowGrayLevelRunEmphasis | 8.2e+12 (0.12,5.6e+26) | 0.067 |
| glrlm ShortRunLowGrayLevelEmphasis | 2.6e+15 (0.05,1.2e+32) | 0.070 |
| firstorder Variance | 1.00 (1.00,1.00) | 0.081 |
| shape LeastAxisLength | 0.97 (0.93,1.00) | 0.082 |
| glszm SmallAreaHighGrayLevelEmphasis | 0.99 (0.98,1.00) | 0.083 |
| glcm ClusterTendency | 0.96 (0.91,1.01) | 0.087 |
| ngtdm Complexity | 1.00 (1.00,1.00) | 0.089 |
| firstorder Energy | 1.00 (1.00,1.00) | 0.091 |
| gldm LargeDependenceLowGrayLevelEmphasis | 1.63 (0.92,2.89) | 0.091 |
| glcm MaximumProbability | 1.8e+04 (0.20,1.7e+09) | 0.093 |
| glszm LowGrayLevelZoneEmphasis | 1.8e+07 (0.06,5.3e+15) | 0.093 |
| gldm SmallDependenceLowGrayLevelEmphasis | 2.3e+86 (1.7e-15,3.2e+187) | 0.094 |
| glrlm GrayLevelVariance | 0.88 (0.77,1.02) | 0.095 |
| glcm Imc1 | 3.3e+03 (0.24,4.5e+07) | 0.096 |
| gldm GrayLevelVariance | 0.88 (0.76,1.02) | 0.10 |
| glcm ClusterProminence | 1.00 (1.00,1.00) | 0.11 |
| gldm DependenceNonUniformity | 1.00 (1.00,1.00) | 0.11 |
| glrlm RunLengthNonUniformity | 1.00 (1.00,1.00) | 0.11 |
| glcm SumSquares | 0.88 (0.75,1.03) | 0.11 |
| firstorder TotalEnergy | 1.00 (1.00,1.00) | 0.12 |
| glcm DifferenceVariance | 0.79 (0.58,1.07) | 0.12 |
| glcm JointEnergy | 6.6e+06 (0.01,3.6e+15) | 0.13 |
| glszm GrayLevelNonUniformity | 1.00 (1.00,1.00) | 0.13 |
| shape VoxelVolume | 1.00 (1.00,1.00) | 0.13 |
| shape MeshVolume | 1.00 (1.00,1.00) | 0.13 |
| shape Flatness | 0.08 (2.5e-03,2.40) | 0.14 |
| glcm Contrast | 0.91 (0.80,1.03) | 0.15 |
| shape SurfaceArea | 1.00 (1.00,1.00) | 0.15 |
| glcm DifferenceEntropy | 0.40 (0.11,1.41) | 0.16 |
| shape MinorAxisLength | 0.98 (0.95,1.01) | 0.16 |
| firstorder Maximum | 1.00 (1.00,1.00) | 0.16 |
| glrlm LongRunLowGrayLevelEmphasis | 967.20 (0.07,1.3e+07) | 0.16 |
| glcm DifferenceAverage | 0.55 (0.23,1.32) | 0.18 |
| firstorder 90Percentile | 1.00 (0.99,1.00) | 0.19 |
| **IHC MMR (Intact vs abnormal)** | **OR (95% CI)** | **p-value** |
| glcm Imc1 | 2.7e-05 (9e-12,81.00) | 0.17 |
| gldm SmallDependenceHighGrayLevelEmphasis | 0.97 (0.93,1.02) | 0.19 |
| **LVSI (Positive or indeterminate vs absent)** | **OR (95% CI)** | **p-value** |
| shape Flatness | 0.09 (4.5e-03,1.93) | 0.13 |
| glcm InverseVariance | 1.1e-03 (1.3e-07,8.91) | 0.14 |
| firstorder Kurtosis | 1.40 (0.89,2.22) | 0.15 |
| glcm Imc2 | 12.88 (0.40,413.22) | 0.15 |
| glszm ZonePercentage | 60.44 (0.19,1.9e+04) | 0.16 |
| gldm DependenceNonUniformityNormalized | 3.2e+04 (0.01,7.3e+10) | 0.17 |
| firstorder Skewness | 2.26 (0.67,7.57) | 0.19 |
| shape Maximum2DDiameterRow | 0.99 (0.97,1.01) | 0.19 |
| glrlm RunVariance | 1.64 (0.78,3.46) | 0.19 |
|  |  |  |
